# Gender-based violence and infectious disease in humanitarian settings: lessons learned from Ebola, Zika, and COVID-19 to inform syndemic policy making

**DOI:** 10.1186/s13031-021-00419-9

**Published:** 2021-11-20

**Authors:** Melissa Meinhart, Luissa Vahedi, Simone E. Carter, Catherine Poulton, Philomene Mwanze Palaku, Lindsay Stark

**Affiliations:** 1 Brown School at Washington University in St. Louis, One Brookings Drive, Campus Box 1196, St. Louis, MO 63130 USA; 2 UNICEF, Public Health Emergencies, Geneva Avenue de la Paix 5 - 7, 1202 Genève, Switzerland; 3 UNICEF, United Nations Plaza, New York, NY 10017 USA; 4 UNICEF, 372, Av. Col. Mondjiba, Kinshasa, Ngaliema Democratic Republic of the Congo

## Abstract

**Background:**

The impacts of infectious disease outbreaks, epidemics, and pandemics are not gender neutral. Instead, infectious diseases and gender-based violence (GBV) mutually reinforce each other. Women and girls in humanitarian settings are disproportionately impacted as crises exacerbate gender inequality, violence, and community transmission. A syndemic model of infectious disease and GBV draws attention to their critical linkage, enabling more effective approaches to address both infectious disease transmission and GBV prevalence.

**Main body:**

Implementation of infectious disease control measures have been consistently absent of critical gender considerations in humanitarian settings. We drew learnings from Ebola, Zika, and COVID-19 to highlight how women and girls living in humanitarian settings have faced bi-directional syndemic vulnerabilities between GBV and infectious disease. Our findings indicate that Ebola, Zika, and COVID-19 exacerbated GBV risk and experience of GBV increased community transmission of these infectious diseases. Moreover, we identified a failure of existing policies to address this mutually deleterious linkage. Thus, we advocate for policymakers to ask three foundational questions: (i) *What* are the gendered bi-directional risk pathways between infectious disease and GBV?; (ii) *How* can we act on the gendered risk pathways?; and, (iii) *Who* should be involved in designing, implementing, and evaluating gender-sensitive policies?

**Conclusion:**

Our syndemic policy framework challenges existing thinking on a neglected issue that disproportionally impacts women and girls. By offering foundational guidance to address and thwart the syndemic of infectious disease and GBV in humanitarian settings, we endeavor to proactively and holistically address the reinforcing linkage between GBV and current or emergent infectious diseases.

## Introduction

Emergent literature explores syndemic relationships between gender-based violence (GBV) and COVID-19, wherein each exacerbates risk of the other [1–3]. Defined as violence perpetrated against someone based on their gender expression or identity, GBV includes intimate partner violence, deprivation, economic violence, sexual violence, child marriage, sexual abuse and exploitation, and reproductive coercion [4]. GBV disproportionately threatens the safety and wellbeing of women/girls globally and is heightened in humanitarian settings [4–6].

Risk factors for GBV are magnified during infectious disease outbreaks [1, 3, 7]. For instance, epidemic or pandemic control policies that enforce lockdown measures can heighten socio-economic precarity and the feminization of poverty: known risk factors for transactional sex and sexual abuse and exploitation [8, 9]. In turn, GBV can influence community transmission of infectious disease if public health policies fail to consider how gender norms and gender inequality intersect with the chain(s) of transmission [2]. For example, sexual violence may increase the risk of infectious disease exposure when pathogens are transmitted sexually [10].

Originating from medical anthropology, syndemics occur when: (i) two or more diseases or adverse health conditions cluster within a population; (ii) political and social factors impact the clustering of diseases or adverse health conditions; (iii) the clustering of diseases or adverse health states result in adverse social, biological, or behavioral interactions, that heighten a population’s vulnerability and health burden [11, 12]. Thus, the linkage between epidemics exacerbates harm beyond what would otherwise arise if either epidemic occurred in isolation.

Humanitarian settings perpetuate the clustering and interaction of infectious disease and GBV due to the ubiquity of violence, weakening of social support networks, exacerbation of patriarchal norms, and breakdown of health, economic, social, and political systems [1, 4]. An examination of syndemic relationships between recent outbreaks of infectious diseases and GBV in humanitarian settings reveals the structural factors that underpin vulnerability and magnify the population health burden. A syndemic lens is critical to developing policies that address both GBV and infectious disease in a manner that sustains progress on gender mainstreaming and transformation [3].

In this paper, we draw on syndemic relationships between GBV and Zika, Ebola, and COVID-19 in humanitarian settings to identify lessons learned that can inform gender-sensitive public health policies. We explore the bi-directional relationships between infectious diseases and GBV to illustrate how their dynamic interplay magnifies the population health burden. We then present a policy framework informed by syndemic theory, to better address the intersections between infectious disease and GBV.

## Infectious disease outbreaks and GBV: exploring bi-directional syndemic relationships

The implementation of infectious disease control measures, absent of critical gender considerations, has situated GBV as an afterthought of public health response [13]. Consequently, women and girls living in humanitarian settings have faced bi-directional syndemic vulnerabilities between GBV and at least three major infectious diseases in recent history: Ebola (2013-present), Zika (2015–2016), and COVID-19 (2020-present).

### Linking infectious disease outbreaks with GBV: consequences for women and girls

Negative consequences of gender insensitive pandemic control policies with respect to GBV include increasing the proximity of survivors/victims to abusers, magnifying household socio-economic strain, reinforcing household gender roles, exacerbating inadequate access to sexual/reproductive health services, and limiting gender-specific data collection and analyses; all these consequences compound in humanitarian settings and are exacerbated for women and girls.

As illustrated by the COVID-19 pandemic, in the absence of widespread vaccination and effective pharmacological treatment, governments have implemented ad-hoc stay-at-home orders and regional lockdowns. In humanitarian settings, the breakdown of health and social services combined with lockdowns have culminated in inadequate protections for women and girls, as well as elevated levels of domestic and intimate partner violence [7, 14]. Prolonged stay-at-home orders and regional lockdowns during COVID-19 raise household economic precarity and stress which are known risk factors for domestic and intimate partner violence [15]. These stay-at-home orders and regional lockdowns also disproportionately hinder girls’ educational attainment which is a risk factor for child marriage [7, 16].

During the Ebola epidemics in the Democratic Republic of the Congo (DRC) and West Africa, public health policies failed to adequately consider the gendered division of household labor. Adherence to handwashing and sanitation measures elevated the demand for household water supplies and thus increased the frequency with which women and girls left the home to collect water [17]. Traveling long distances to gather sufficient water supplies during periods of increased demand elevated the risk of experiencing sexual violence and harassment from opportunistic perpetrators, especially when conflict and civil unrest were present [13, 17, 18].

Moreover, infectious disease control policies rarely consider GBV and sexual/reproductive health services as essential, thereby resulting in a consistent erosion of services in humanitarian settings [19–21]. The challenges faced by pregnant survivors of sexual violence with respect to accessing psycho-social support, healthcare, and abortion are magnified by pandemics and epidemics. The lack of sexual and reproductive health services during COVID-19 is particularly concerning given the elevated rates of unintended pregnancies [19] and unsafe abortions [22]. The lack of sexual and reproductive healthcare combined with wide-spread fear of bodily fluids during the West Africa and Eastern DRC Ebola outbreaks resulted in young women being shamed during menstruation or childbirth and subsequently sent to Ebola Treatment Centers [23].

The Zika epidemic in South America also draws attention to the critical need for sexual/reproductive healthcare. A fetus developing during the mother’s period of Zika virus infectivity faced an increased risk of developing congenital defects such as microcephaly [23, 24]. Women who gave birth to children with microcephaly during the Zika epidemic experienced elevated paternal abandonment [25], resulting in single motherhood and the potential for socio-economic adversity. Moreover, campaigns encouraging women to postpone pregnancy fail to consider lack of reproductive autonomy, inadequate access to contraception, and in some contexts criminalized abortion, particularly with respect to Zika-affected pregnancies resulting from sexual violence. Thus, gender-insensitive infectious disease control measures deepen both individual and structural-level GBV, thereby further eroding progress on gender equity in humanitarian settings. Women and girls in humanitarian settings experience pandemics and epidemics within a context of heightened GBV and reduced service provision.

### GBV and sustained community transmission: consequences for infectious disease control

The relationship between COVID-19 and GBV is not unidirectional: individual and structural-level GBV increase the risk for sustained community transmission. One pathway from GBV to increased community transmission is through the sexual transmission of infectious disease. Even in the absence of epidemics or pandemics, access to and negotiation of contraceptives is negatively impacted by the structural gender inequality present in humanitarian settings. Unprotected sexual intercourse may increase the risk of exposure to certain pathogens. Sexual transmission of infectious disease may be heightened when transactional sex is used as a means to alleviate socio-economic strain. For instance, given that Ebola virus RNA can persist in semen for prolonged periods, unprotected sexual intercourse through sexual violence, transactional sex, and sexual abuse and exploitation propagated community transmission [10, 26]. The sexual transmission of Ebola, including through sex work and sexualized violence, not only introduced the virus into households but also spread the virus between communities [13].

Within a wider context of limited sexual/reproductive health and rights [27], sexual-violence perpetrated by Zika-positive perpetrators could result in infection alongside pregnancy and fetal congenital defects. Further compounding vulnerabilities was the exclusion of pregnant and lactating women from the development and administration of Zika vaccine trials [24]. Thus, sexual violence rendered women and girls vulnerable to Zika infection while those who were pregnant and lactating were systematically excluded from vaccination.

In Brazil, men in urban areas who engaged in casual sexual encounters during the epidemic were more likely to be Zika positive, making them potential carriers of the virus given that Zika virus RNA can remain in semen for prolonged periods [28, 29]. Thus, victims of sexual violence, sex workers, and those who participated in transactional sex may contract Zika through sexual contact with positive men, particularly if knowledge about Zika’s sexual transmission is inadequate [30, 31].

Further, emerging evidence indicates that some abusers are leveraging the uncertainty and fear associated with the COVID-19 pandemic to further assert power and control by engaging in distinct forms of psychological intimate partner violence: threatening to infect the victim, reducing access to hygiene supplies, and limiting access to testing and vaccination [30, 31]. In the absence of GBV prevention and response services, psychological forms of intimate partner violence could increase exposure to COVID-19 through limiting access to needed hygiene supplies and healthcare [32, 33].

In the DRC and West Africa, Ebola’s chain of transmission intersected with household gender norms, as women and girls led caretaking responsibilities for family members who were Ebola positive [13, 17]. Due to widespread morbidity and mortality, such unpaid and often unrecognized gendered familial responsibilities were heightened during the Ebola epidemic and increased the risk of women and girls coming into contact with infected bodily fluids [13, 17]. To further magnify the gendered burden, even when made available, personal protective equipment may not adequately protect women or girls because sizes are designed according to the dimensions of men [34]. In the absence of household water, soaps, adequate personal protective equipment, and disinfectants, exposure to contaminated bodily fluids while caregiving contributes to gendered viral transmission.

Additionally, within households in humanitarian settings access to technology such as mobile phones, TV, radio, and internet connection, is limited. For example, one phone many be shared among members of the entire household. Due to gender discrimination, women and girls face reduced access to and familiarity with digital tools [35, 36]. The gender digital divide—referring to the disproportionate lack of digital skills, permitted use, and access to technology among women and girls—can inhibit access to lifesaving public health information pertaining to infectious disease testing, routes of transmission, and vaccination [3, 35].

The failure to recognize and act on direct and indirect pathways of community transmission associated with GBV leaves women and girls vulnerable to infectious disease exposure. In fact, the failure to act on previous evidence from infectious diseases to safeguard the rights and health of women and girls is a form of structural GBV.

## Building a syndemic policy framework

**Fig. 1:**
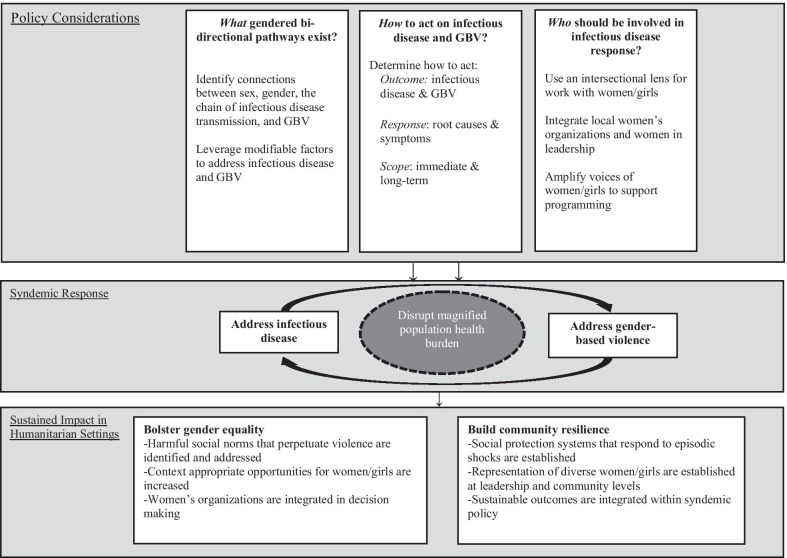
Conceptual model for syndemic policy framework addressing infectious disease and GBV

A syndemic understanding highlights the mutually reinforcing and bi-directional relationship between infectious disease and GBV and highlights the urgent need for policy reform. Building on existing evidence, we propose three considerations to support national and multi-lateral syndemic policies. Each consideration centralizes the importance of utilizing or collecting gender sensitive data to inform inclusive policies that address the unique risks experienced by women and girls. To ground these considerations, we integrate examples and lessons learned from an operational and integrated analytics unit that has been embedded to support the DRC’s Ministry of Health since the 2018 Ebola outbreak; the Social Sciences Analytics Cell (CASS—Cellule D’Analyse en Sciences Sociales) seeks to bring together epidemiological, social sciences, health services, programmes and other data to better understand outbreak dynamics and inform public health and outbreak response [37, 38]. To simultaneously bolster gender equity and community resilience while also addressing the proliferation of GBV and infectious disease, we encourage policymakers to ask the following questions: (i) *What* are the gendered bi-directional risk pathways between infectious disease and GBV; (ii) *How* can policymakers act on the gendered risk pathways?; and, (iii) *Who* should be involved in designing, implementing, and evaluating gender-sensitive policies? (Fig. 1).

## *What* are the gendered bi-directional risk pathways between infectious disease and GBV?

Policy makers should first determine what risk pathways exist between infectious disease and GBV. Central to building syndemic policies is understanding how the chain of infectious disease transmission intersects with biological sex, gender, and GBV. Interdisciplinary teams of infectious disease experts, epidemiologists, social scientists, public health officials, gender specialists, and local women’s organizations should use their expertise and gender-sensitive data to map the gendered pathways of community transmission, think critically about how risk pathways impact people differently based on gender and sex, and identify contextually appropriate critical leverage points to protect against both infectious disease and GBV.

At the onset of the Ebola outbreak in Eastern DRC in October 2018, epidemiological data indicated that more women were being affected by Ebola than man. Vaccination data, however, were not disaggregated by sex, nor did the vaccination data provide information on those who were not eligible for the vaccine because they were pregnant and/or breastfeeding. Thus, the CASS sought to better understand risk factors affecting women. Through their research, the CASS found that pregnant and/or breastfeeding women who had been identified through contact tracing were not included in epidemiological surveillance and psycho-social follow up due to their vaccine ineligibility [37]. The study also found that surveillance forms did not specify whether a woman was pregnant and/or breastfeeding, reducing the ability to identify and support women who had become high risk. Particularly concerning, many breastfeeding women working in high risk healthcare facilities were not vaccinated or chose to stop breastfeeding in order to become vaccine eligible. Not vaccinating or providing follow up support to pregnant and/or lactating women placed them at a greater risk of infecting others. The CASS’s evidence resulted in an immediate change to documenting women who were not eligible for the vaccine, via surveillance and vaccination data. Moreover, breastfeeding women who were frontline responders were offered support kits if they chose to stop breastfeeding to access the vaccine. In June 2019, the Strategic Advisory group eventually implemented a change in policy to allow access to the Ebola vaccine trial for pregnant and/or breastfeeding women.

When available for analysis or feasible to collect, gender-sensitive data is critical to advancing knowledge of bi-directional risk pathways between infectious disease and GBV [2, 39]. Gender-sensitive data collection involves not only sex disaggregated data, but also collecting data on contextual gender norms For example,, gender norms of domestic labor and caregiving can increase the risk of women and girls coming in contact with infectious bodily fluids or respiratory droplets when caring informally for sick kin. Moreover, vertical transmission from the mother to the fetus infection intersect with women’s limited reproductive autonomy and rights. Lastly, viral transmission through breastfeeding is an important biological sex consideration, as mothers may have to carefully weigh the risks of breastfeeding (i.e. viral transmission) and formula feeding (e.g. access, costs, and water quality).

A separate critical understanding is how commonly implemented public health measures, such as national lockdown, pose adverse effects on socio-economic status that disproportionately affect women and girls in terms of GBV. For example, economic lockdowns result in greater gender pay disparities and financial strain within households: known drivers of intimate partner violence [40, 41]. Financial strain can also increase the vulnerability of women and girls to sexual exploitation and abuse and transactional sex [8, 9]. Given the exacerbated economic strains during infectious disease outbreaks, syndemic policies should integrate mechanisms through which women may be financially supported, such as paid participation in response planning and intentionally hiring women’s associations to support basic production (e.g. masks or soaps) [38].

By collecting and utilizing gendered data to map such bi-directional risk pathways, policymakers can identify actionable target areas that disrupt the connection between infectious disease, GBV, and gender inequality. To address the humanitarian context in which the bi-directional pathways are occurring, robust social protection systems can improve economic opportunities and mitigate drivers of GBV [42, 43].

## *How* can policymakers act on the gendered risk pathways?

After identifying bi-directional syndemic pathways, policymakers should consider how to act most effectively by considering whether policies: (i) address root causes and/or symptoms of the syndemic, and (ii) have immediate and/or long-term impact. The importance of gender data extends to these decisions as policymakers must understand the gendered nature of the syndemic in order to inclusively respond and evaluate.

Social determinants of women’s health and sustainable public health infrastructure are two root causes of the syndemic that can be addressed by gender-sensitive polices. In terms of identifying the root causes of syndemic risk pathways, pertinent social determinants of health in humanitarian settings include loss of human rights, limited economic opportunity, and sustained exposure to stressors [44]. Thus, syndemic policies should consider the role of such social determinants of health during outbreaks, epidemics, and pandemics, and identify how they uniquely impact women and girls in humanitarian settings. Based on evidence of the socio-economic impact of Ebola [38], UNICEF intentionally hired women’s organizations during COVID-19 to support mask making in the DRC.

Also of critical importance, responsive public health infrastructure are a cornerstone for service provision, the early detection of epidemics, rapid surveillance of infectious disease spread, and control of population movement. In response to a request by the Ministry of Health in 2019, the CASS researched why many households and communities were not participating in decontamination practices. Findings demonstrated the reluctance by community healthcare providers was largely driven by the fact that decontamination efforts were implemented by external teams, which embedded distrust within the community [37]. Changes were made to the strategic response plan to involve community members in decontamination efforts and to train men and women from the community in infection prevention control.

Additionally, syndemic policies should consider both immediate and long-term approaches. Humanitarian response is often hindered by funding cycles that prioritize short-term results despite calls for sustainable solutions [45]. Syndemic responses can promote the long-term socio-economic advancement of women and girls. Public health responses in humanitarian settings have often integrated men in paid opportunities; the responsibility of unpaid work, such as sensitization campaigns and reporting illness and exposure to pathogens within the home, disproportionately falls on women [46]. Long-term syndemic response should consider providing direct economic opportunities to women, especially women-led and home-based businesses. In addition to supporting local economies, advancing socio-economic opportunity for women and girls in a contextually sensitive manner may also reduce vulnerability to GBV. Syndemic response can also invest in shifting harmful social norms that perpetuate gender inequality and exacerbate GBV. As another example from the CASS, communication strategies were implemented to describe the various ways in which Ebola resurgence can occur in order to mitigate the disproportionate blame of Ebola’s sexual transmission that was previously placed on women in Butembo [38].

## *Who* should be involved in designing, implementing, and evaluating syndemic?

Lastly, policy makers should consider who is best situated to design, implement, and evaluate syndemic policies. Participatory approaches, led by women and girls from the community, that explore gender norms which perpetuate GBV and community transmission are a cornerstone to addressing syndemic vulnerabilities. In support of the 2021 Ebola resurgence in Butembo DRC, the CASS worked with response teams to review the 55 studies and 112 co-developed recommendations from the 2018–2020 outbreak [38]. This included critically addressing the many existing evidence based recommendations related to the inclusion of women and girls in the response. The actions taken included: ensuring women’s active participation in the resurgence response through hiring committees to mitigate risks of sexual exploitation during recruitment, and collaboration with women’s organizations to increase women’s participation in informing response activities [38]. By hiring women to both inform and participate in the Ebola response activities in Butembo, the outbreak response in the DRC was already familiar with syndemic thinking prior to COVID-19 and better prepared to addressed the gendered influence of COVID-19.

Women and girls are not monolithic; gendered risk pathways of infectious disease also intersect with other forms of oppression such as ableism, racism, and religious persecution [4]. To be contextual, policymaking must integrate diverse perspectives from women and girls who face multiple intersectional risks for GBV and infection [39, 47]. While not without its own challenges in regard to inclusivity, the CASS has prioritized the integration of different perspectives in its efforts to provide evidence on the negative impacts of COVID-19 Public Health and Social measures, particularly regarding its harmful impact on the safety and health of women and girls [37].

Originating from gender theory, intersectionality is a framework for understanding that people experience intersecting forms of oppression, discrimination and marginalization based on their co-existing identities [48]. Evidence from COVID-19 indicates that persons with marginalized sexual orientations and gender identities have reduced access to services and elevated risks for GBV [49, 50]. The integration of women and girls, as well as persons with marginalized sexual orientations and gender identities, in the design and implementation of policies is not merely a matter of filling quotas to achieve parity. Infectious disease control policies that are devoid of evidence-based considerations for how gender impacts the navigation of humanitarian settings cannot adequately mitigate community transmission risk factors. There is a population cost both in terms of economics and health to unpaid labor, intimate partner violence, and unmet sexual/reproductive health needs.

Centering the voices of women and girls may also help to identify which humanitarian and medical responders are best positioned to screen for GBV victimization and perpetration, as inform better GBV training for public health responders. Also important is to identify models of GBV service provision that do not perpetuate community transmission. Instead of the default response, to reduce humanitarian GBV services during pandemics, GBV services should expand to include syndemic prevention and response considerations and adjust to mitigate against community transmission. To amplify the voices of women from communities, the CASS prioritized two-way communication during Ebola whereby women’s associations provided study contributions to inform weekly evidence briefings at coordination and sub coordination levels, and the CASS returned to women’s organizations to share how their evidence is informing decision making [37].

Women and girls should be involved in planning and implementation to examine how services can be safely accessed without increasing the risk of community transmission or GBV. Remote service delivery, such as virtual safe spaces, provides an innovative avenue to engage with women and girls [51]. After rigorous pilot testing for safety and privacy, digital technology may emerge as lifesaving programmatic tools that can increase safe and informative program access to women and girls in humanitarian settings. Similar considerations should be made to review under what conditions programming for men and boys, such as healthy masculinity programs, can be conducted remotely through technology. Moreover, consultations can help to better tailor gender transformative public messaging that can be implemented in parallel with infectious disease public health information propagated through mass media.

## Conclusion

Solely addressing the threat of infectious disease in humanitarian settings will not address the full spectrum of syndemic vulnerabilities [3]. We advocate for policymakers to integrate a gender-sensitive policy framework that addresses the syndemic of infectious disease and GBV. Accordingly, we encourage policymakers to ask three foundational questions: (i) *what* are the bi-directional risk pathways between infectious disease and GBV?; (ii) *how* can we act on them?; and (iii) *who* should be involved? In doing so, policymakers will safeguard the rights and health of women and girls in humanitarian settings during infectious disease outbreaks, epidemics, and pandemics, while also bolstering gender equality and community resilience. Building on the 2030 Agenda for Sustainable Development “leave no one behind” commitment [52] and the work of the gender and COVID-19 project regarding how to design gender-responsive pandemic plans [39], this syndemic policy guidance offers a policy framework to proactively and holistically address the reinforcing relationship between GBV and current as well as emergent infectious diseases.

## Data Availability

Not applicable.

## Abbreviations

CASS: Cellule D’analyse en Sciences Sociales/Social Sciences Analytics Cell
DRC: Democratic Republic of the Congo
GBV: Gender-based violence
